# The Emerging Functions of LRRK2 and Rab GTPases in the Endolysosomal System

**DOI:** 10.3389/fnins.2020.00227

**Published:** 2020-03-18

**Authors:** Tomoki Kuwahara, Takeshi Iwatsubo

**Affiliations:** Department of Neuropathology, Graduate School of Medicine, The University of Tokyo, Tokyo, Japan

**Keywords:** LRRK2, lysosome, endosome, Rab, phosphorylation, trafficking

## Abstract

The leucine-rich repeat kinase 2 (*LRRK2*), the most common causative gene for autosomal-dominant familial Parkinson’s disease, encodes a large protein kinase harboring multiple characteristic domains. LRRK2 phosphorylates a set of Rab GTPases in cells, which is enhanced by the Parkinson-associated LRRK2 mutations. Accumulating evidence suggests that LRRK2 regulates intracellular vesicle trafficking and organelle maintenance including Golgi, endosomes and lysosomes. Furthermore, genetic knockout or inhibition of LRRK2 cause lysosomal abnormalities in rodents and primates, and cells from Parkinson’s patients with LRRK2 mutations also exhibit altered lysosome morphology. Cell biological studies on LRRK2 in a diverse cellular context further strengthen the potential connection between LRRK2 and regulation of the endolysosomal system, part of which is mediated by Rab phosphorylation by LRRK2. We will focus on the latest advances on the role of LRRK2 and Rab in relation to the endolysosomal system, and discuss the possible link to the pathomechanism of Parkinson’s disease.

## Introduction

Mutations in leucine-rich repeat kinase 2 (*LRRK2*) gene cause late-onset, autosomal-dominant forms of Parkinson’s disease (PD) (Paisan-Ruiz et al., 2004; Zimprich et al., 2004). To date, at least seven missense mutations (N1437H, R1441C/G/H, Y1699C, G2019S, I2020T) have been identified as definitely causal, and G2019S is the most frequent mutation among them. The pathology of PD is characterized by the loss of midbrain dopaminergic neurons as well as the formation of Lewy bodies, the cytoplasmic inclusion composed primarily of α-synuclein filaments. Importantly, a majority of familial PD patients harboring LRRK2 mutation display an accumulation of Lewy bodies in affected brain lesions, although a range of heterogeneity (i.e., some cases are Lewy body predominant, while others exhibit tau deposits or lack specific intraneuronal inclusions) characterizes the neuropathology of LRRK2 mutant PD (Khan et al., 2005; Kalia et al., 2015). The link of LRRK2 to sporadic PD has also been suggested by a set of genome-wide association studies (GWAS) where common variants around *LRRK2* gene have been identified as a risk factor of PD (Satake et al., 2009; Simón-Sánchez et al., 2009; Lill et al., 2012). In addition, activation of LRRK2 kinase has been implicated in sporadic PD and non-LRRK2 PD models (Di Maio et al., 2018), placing LRRK2 in more common pathway for PD manifestation. Thus, elucidating the role of LRRK2 in pathological as well as physiological situations may provide hints for the establishment of rational strategy to treat PD.

In addition to PD, previous GWAS have also identified *LRRK2* in a susceptible locus for Crohn’s disease (Barrett et al., 2008) and leprosy (Zhang et al., 2009), both of which are immune-related disorders. Some functional variants in *LRRK2* gene influencing the disease risk are shared between Crohn’s disease and PD (Hui et al., 2018). Another study has also pointed to a genetic association between LRRK2 and susceptibility to systemic lupus erythematosus (SLE) (Zhang et al., 2017). Consistently, LRRK2 is considered to be involved in a wide range of disorders affecting both brain and periphery.

LRRK2 is a multidomain protein kinase harboring several characteristic domains, such as ankyrin repeats, LRR (leucine-rich repeat), ROC (Ras of complex), COR (*C*-terminal of ROC), WD40 and kinase domains. Due to the presence of a tandem ROC-COR domain, LRRK2 is classified as a member of the ROCO protein family (Bosgraaf and Van Haastert, 2003). LRRK2 expression is detected in a broad range of organs and tissues including brain, and is especially high in kidney, lung and spleen (Biskup et al., 2007; Li et al., 2007; Maekawa et al., 2010) as well as in immune cells (Gardet et al., 2010; Maekawa et al., 2010). In the central nervous system, LRRK2 is expressed in a subset of neurons including those in the substantia nigra (Biskup et al., 2006; Hatano et al., 2007), but is reported to be more highly expressed in astrocytes and microglia (Henry et al., 2015). In immune cells, LRRK2 expression is especially high in macrophages, B cells and neutrophils (Biskup et al., 2007; Li et al., 2007; Gardet et al., 2010; Maekawa et al., 2010; Fan et al., 2018). A noteworthy finding is that the expression of LRRK2 in macrophages is potently induced by IFN-γ stimulation (Gardet et al., 2010). These expression patterns point to variable roles of LRRK2, such as immune-related functions.

Within cells, LRRK2 is known to be predominantly distributed throughout the cytoplasm (West et al., 2005), whereas biochemical fractionation studies have shown that at least a portion is associated with membranes, suggesting the localization to specific organelles or membrane microdomains (Hatano et al., 2007; Berger et al., 2010; Schapansky et al., 2014). However, immunocytochemical or ultrastructural analyses have not provided consistent results for the LRRK2 localization; the possible subcellular locations include Golgi, mitochondria, endosomes, lysosomes, endoplasmic reticulum (ER), multivesicular bodies, amphisomes and autolysosomes (Biskup et al., 2006; Hatano et al., 2007; Alegre-Abarrategui et al., 2009; Vitte et al., 2010). We have detected the endogenous LRRK2 on a portion of enlarged lysosomes, which was observed in ∼0.1-1% of total healthy cells or in a majority of cells treated with chloroquine, by using three well-characterized antibodies (Eguchi et al., 2018). In any event, the following issues should be taken into account when interpreting the localization studies; first, overexpressed proteins often display non-physiological localization patters, and indeed LRRK2 tends to form aggregate- or skein-like structures in cells when overexpressed in cultured cells. Another issue is that, even when endogenous LRRK2 are analyzed by specific antibodies, their properties on immunocytochemical analyses are not necessarily defined. The endolysosomal localization of LRRK2 will be specifically discussed later in this article.

Endolysosomal system, especially lysosomes, has attracted much attention in the field of LRRK2 research, given the accumulating evidence that knocking out LRRK2 or introduction of pathogenic mutations causes lysosomal abnormalities in animals and cultured cells. In addition, dysregulation of endolysosomal system has been implicated more broadly in familial and sporadic PD other than LRRK2-related PD. For instance, the lysosomal enzyme glucocerebrosidase (GBA) and the lysosomal K^+^ channel TMEM175 are well-validated risk factors identified by GWAS of sporadic PD (Nalls et al., 2014; Chang et al., 2017; Blauwendraat et al., 2019; Iwaki et al., 2019). Also, the lysosomal *P*-type ATPase ATP13A2 (PARK9) and the retromer complex component VPS35 (PARK17) regulating endosome-to-Golgi transport are the products of the causative genes for familial PD or related diseases (Ramirez et al., 2006; Vilariño-Güell et al., 2011; Zimprich et al., 2011). The endolysosomes are further considered to play an important role in the aggregation or propagation of α-synuclein deposited in PD brains.

As the kinase activity of LRRK2 has been shown to be responsible for most of its functions in endolysosomes and other systems, a deeper understanding of the downstream of LRRK2 kinase activity is critical. The substrates of LRRK2 in cells have long been enigmatic until the identification of a set of Rab GTPases (Steger et al., 2016). Small Rab GTPases are the key regulators of intracellular vesicle trafficking, constituting the largest family in the Ras-related small GTPase superfamily. More than 60 different Rabs have been identified in humans, but it is noteworthy that the substrates of LRRK2 are limited to a small proportion, *e.g.*, Rab8 and Rab10 (Steger et al., 2016, 2017). The importance of this phosphorylation is particularly highlighted by the finding that LRRK2 pathogenic mutations commonly augment its activity to phosphorylate these Rab GTPases. Thus, elucidating the role and significance of Rab phosphorylation is vital to understand the pathways leading to PD as well as the basic biology of LRRK2, including those in endolysosomes. In this article, we aim to summarize our current understanding about the relationship among LRRK2, Rab, and endolysosomal system, and discuss the possible involvement of the dysregulation of this system in the pathomechanism of PD.

## The Role of LRRK2 in Lysosomal Homeostasis

Lysosomes are membrane-enclosed organelles that play essential roles in many cellular processes including cell growth, division and differentiation (Pu et al., 2016; Lawrence and Zoncu, 2019), whereas they have classically been established as terminal digestive system degrading materials from both inside and outside of the cells (de Duve, 2005). Lysosomes contain a series of acid-dependent hydrolases as well as highly glycosylated integral membrane proteins. Similar properties are shared with a set of cell type-specific compartments called “lysosome-related organelles,” such as melanosomes and lung lamellar bodies (Dell’Angelica et al., 2000). The relationship between lysosome and LRRK2 has been particularly highlighted over the past years, since a number of studies have reported the lysosomal pathology in *Lrrk2* knockout (KO) animals, such as age-dependent accumulation of autofluorescent lipofuscin granules that are composed of undigested materials derived from lysosomes (Tong et al., 2010, 2012; Herzig et al., 2011; Hinkle et al., 2012; Baptista et al., 2013; Ness et al., 2013; Boddu et al., 2015; Fuji et al., 2015; Kuwahara et al., 2016). Indeed, detailed histopathological analyses have demonstrated a marked enlargement of lysosomes or lysosome-related organelles (called lamellar bodies) in the kidney or lung of *Lrrk2* KO rodents (Herzig et al., 2011; Baptista et al., 2013; Fuji et al., 2015). Treatment with LRRK2 kinase inhibitors of non-human primates also induced abnormal cytoplasmic accumulation of lamellar bodies in type II pneumocytes of the lung (Fuji et al., 2015). Thus, there is little doubt that the physiological function of LRRK2 is related to the maintenance of lysosomal morphology or functions.

The close relationship between LRRK2 and lysosomes has already been described earlier in LRRK2 research. For example, neurons overexpressing pathogenic mutant LRRK2 accumulate phospho-tau-positive lysosomal inclusions (MacLeod et al., 2006), and LRRK2 is localized to membranous and vesicular structures, including lysosomes and endosomes, in mammalian brains (Biskup et al., 2006). Later on, the lysosomal regulation by LRRK2 have been increasingly described using various cellular systems and model organisms. In Drosophila, an ortholog of LRRK2 (Lrrk) localizes to the endolysosomal membranes and negatively regulates Rab7-dependent perinuclear localization of lysosomes (Dodson et al., 2012). In addition, Lrrk loss-of-function flies display the accumulation of markedly enlarged lysosomes that are laden with undigested contents (Dodson et al., 2014). In mouse primary astrocytes, overexpressed LRRK2 localizes primarily to lysosomes and regulates the size of lysosomes through its kinase activity (Henry et al., 2015). Mouse primary neurons harboring LRRK2 G2019S mutation also display altered lysosomal morphology, such as the reduction of lysosomal size and the increase in the number and total area of lysosomes (Schapansky et al., 2018). In our hands, endogenous LRRK2 in mammalian cells negatively regulated the enlargement of overloaded lysosomes (Eguchi et al., 2018), consistent with the above studies. In relation to PD, the disruption of lysosomal morphology was observed in fibroblasts from PD patients harboring the G2019S mutation (Hockey et al., 2015).

The reported effects of LRRK2 on lysosomal morphology *in vivo* or in cultured cells are summarized in Table 1. Knocking out LRRK2 caused lysosomal enlargement in most experiments, whereas the effect of pathogenic mutant LRRK2 (*e.g.*, G2019S) on lysosome size and number is not consistent among studies, which may be due to a variety of experimental conditions including differences in cells/tissues or methods of gene manipulations (overexpression, knockin, etc.). Nonetheless, these studies consistently showed that the effects on lysosomes by LRRK2 is dependent on its kinase activity. Taken together, these studies suggest that LRRK2 kinase plays a pivotal role in the regulation or maintenance of lysosomal homeostasis.

**TABLE 1:** Representative studies on the effect of LRRK2 onlysosome morphology.

References	Cells, tissues	Manipulation	Effects on lysosome morphology
Herzig et al., 2011	mouse kidney, lung	knockout	Increase in size and number of lysosomes in mouse KO kidney proximal tubules and lamellar bodies in KO lung type II cells.
Baptista et al., 2013	rat kidney, lung	knockout	Increase in size and number of lysosomes in rat KO kidney proximal tubules and lamellar bodies in KO lung type II cells.
Dodson et al., 2014	Drosophila	knockout	Enlarged lysosomes with undigested contents in lrrk null flies
Fuji et al., 2015	monkey lung	inhibitor dosing	Increase in size and number of lamellar bodies in the lung of monkey dosed with LRRK2 kinase inhibitors
Henry et al., 2015	Primary mouse astrocytes	overexpression of WT or G2019S, knockout	Lysosomes were enlarged, the number was decreased upon G2019S overexpression. Increase in number in KO cells.
Hockey et al., 2015	G2019S patient fibroblast	endogenous G2019S mutation	Enlarged and clustered lysosomes in LRRK2-PD fibroblasts
Schapansky et al., 2018	Primary mouse neurons	G2019S knockin	Lysosome size was decreased, the number and total area were increased in G2019S neurons.
Eguchi et al., 2018	RAW264.7 cells, HEK293 cells	knockdown, overexpression	Knockdown caused the enlargement upon overload stress. Overexpression, especially PD mutants, suppressed the enlargement.

## LRRK2, Endolysosomal Trafficking and Autophagy

Substances destined for degradation are transported into lysosomes mostly through two distinct processes: the endocytosis of extracellular materials and autophagy of intracellular components. These two processes are dynamically regulated by membrane transport (Saftig and Klumperman, 2009), and LRRK2 has been implicated in both processes. Regarding the endocytosis pathway, Gomez-Suaga et al. (2014) have shown that the overexpression of pathogenic mutant LRRK2 delays endosomal trafficking of the epidermal growth factor receptor (EGFR) by decreasing Rab7 activity-mediated late endosomal budding. Additionally, their recent study has shown that LRRK2-mediated inhibition of Rab8a also is involved in this impaired EGFR trafficking by interfering its recycling (Rivero-Rios et al., 2019).

The affected cargoes are not likely restricted to EGFR, as it has been demonstrated that LRRK2 controls the vesicular endosomal trafficking of major lysosomal membrane proteins (LMPs), such as LAMP1, LAMP2, or LIMP2, to lysosomes through regulation of the adaptor protein complex 3 (AP-3) (Kuwahara et al., 2016). Actually, LRRK2 can bind β3A subunit of the AP-3 complex, and genetic interaction between the orthologs of LRRK2 and AP-3 was revealed in *Caenorhabditis elegans* in terms of the regulation of axon termination. Of note, the endosomal trafficking of LIMP2, a cargo of AP-3 complex, may be particularly important in relation to the pathomechanism of PD, given that LIMP2 is selectively responsible for the intracellular transport of a lysosomal enzyme β-glucocerebrosidase (βGC), a major risk factor for developing PD, to lysosomes through direct binding (Reczek et al., 2007; Saftig and Klumperman, 2009), and that LIMP2 deficiency in mice leads to α-synuclein accumulation as well as the reduction of lysosomal βGC activity (Rothaug et al., 2014). Also, *SCARB2* gene that encodes LIMP2 has been identified at a PD risk locus (Do et al., 2011; Michelakakis et al., 2012; Hopfner et al., 2013), and the recent study of age at onset of PD GWAS that is largest to date has confirmed *SCARB2* as a risk gene (Blauwendraat et al., 2019).

In addition to endocytic pathway, LRRK2 appears to modulate other lytic pathways, such as phagocytosis and autophagy. Regarding phagocytosis, it has been shown that LRRK2 regulates the phagocytic activity in myeloid cells via WAVE2 complex, an actin-cytoskeletal regulator (Kim et al., 2018). Another study has reported that LRRK2 negatively regulates phagosome maturation in macrophages via the recruitment of the Class III phosphatidylinositol-3 kinase (PI3K) complex and Rubicon to the phagosomes (Hartlova et al., 2018). Although both studies clearly showed the involvement of LRRK2 kinase activity, its role in phagocytosis appears to be different; whereas LRRK2 activity facilitates the step of engulfment, it also suppresses phagosomal maturation at a later step.

Regarding autophagy (especially macroautophagy), a lysosome-mediated process of cytoplasmic degradation, a growing number of studies have suggested the involvement of LRRK2. *Lrrk2* KO mice exhibit alterations in the levels of LC3-II and p62, a reliable autophagy marker and an autophagy substrate, respectively (Tong et al., 2010, 2012). *In vitro* studies have shown that the overexpression of G2019S LRRK2 in SH-SY5Y cells caused a marked increase in the LC3-positive autophagic vacuoles (Plowey et al., 2008), and the expression of LRRK2 in HEK293 cells also caused a similar increase in autophagosome formation (Gómez-Suaga et al., 2012). Knockdown of LRRK2 in cells stably expressing fluorescence-tagged LRRK2 increased autophagic activity and prevented the starvation-induced cell death (Alegre-Abarrategui et al., 2009), and the pharmacological inhibition of LRRK2 kinase activity similarly stimulated macroautophagy (Manzoni et al., 2013). In contrast, another study showed that knockdown of endogenous LRRK2 in macrophage or microglial cells decreased LC3-II levels and autophagy flux (Schapansky et al., 2014). Thus, it is not necessarily clear whether LRRK2 facilitates or suppresses the autophagy, and the mechanism of autophagy regulation by LRRK2 remains undefined.

**FIGURE 1:**
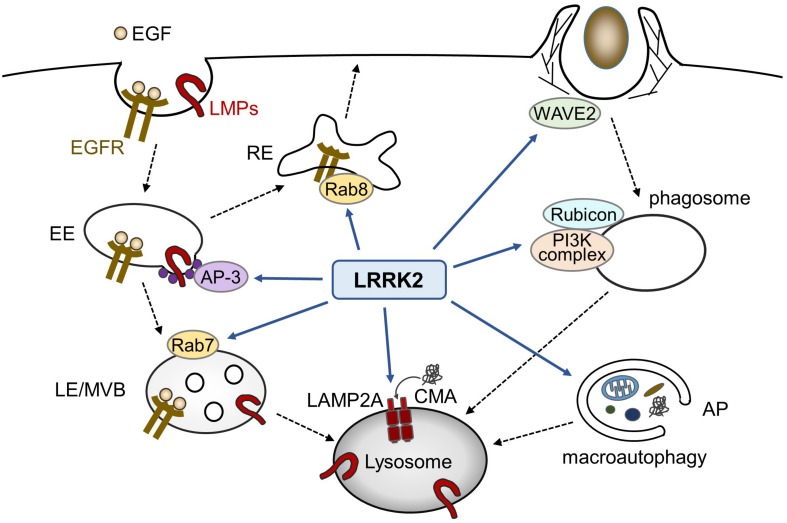
Possible roles of LRRK2 in endolysosomal trafficking. In endocytic pathways, LRRK2 influences the endosomal trafficking of EGFR as well as lysosomal membrane proteins (LMPs). In phagocytosis in myeloid cells, LRRK2 modulates phagocytic activity via WAVE2 complex or phagosome maturation via PI3K complex and Rubicon. LRRK2 has also been reported to regulate macroautophagy and chaperon-mediated autophagy (CMA). EE, early endosome; LE, late endosome, MVB, multivesicular body; RE, recycling endosome; AP, autophagosome.

In addition to macroautophagy, LRRK2 has been shown to be associated with the chaperon-mediated autophagy (CMA); whereas LRRK2 serves as a substrate of CMA, binding of PD-associated mutant LRRK2 with lysosomes in the presence of other CMA substrates adversely results in a defective CMA (Orenstein et al., 2013). Taken together with the data related to endocytosis and phagocytosis, LRRK2 appears to function at diverse steps of lytic processes involving lysosomes (Figure 1).

## The Impact of Rab Phosphorylation by LRRK2

Since LRRK2 kinase activity is considered as a key in the pathomechanisms of PD, much effort has been devoted to the identification of its substrates. The examples of reported cellular substrates include Endophilin A and ribosomal protein S15 (Matta et al., 2012; Martin et al., 2014), although further studies are warranted to validate the phosphorylation of these potential substrates. In 2016, Steger et al. (2016) have reported a subset of Rab GTPases as substrates of LRRK2 in cells. The subsequent and systematic analyses demonstrated that Rab3a-d, Rab5a-c, Rab8a/b, Rab10, Rab12, Rab29 (also known as Rab7L1), Rab35 and Rab43 are phosphorylated by LRRK2 at least upon overexpression (Steger et al., 2017). Other groups have also reported that Rab8, Rab10 and Rab29 behave as excellent substrates of LRRK2 in cells (Fujimoto et al., 2018; Liu et al., 2018; Madero-Perez et al., 2018a). At endogenous levels, LRRK2-mediated phosphorylation likely occurs on Rab3a-d, Rab8a/b, Rab10, Rab12, Rab35 and Rab43 (Steger et al., 2017). Phosphorylation site is located in the middle of switch II region of Rab GTPases, *e.g.*, Thr72 in Rab8a, and the structurally equivalent sites in other Rabs, which is predicted to undergo a conformational change upon GTP/GDP binding. Notably, another study have reported that LRRK1, a paralog of LRRK2, phosphorylates Rab7 at Ser72 (Hanafusa et al., 2019), suggesting a strong functional connection between LRRK and the Rab family proteins.

Recent advances in the analysis of phosphorylation owes a great deal to the development of Phos-tag SDS-PAGE technique (Kinoshita et al., 2006). Researchers no longer need to raise phospho-specific antibodies but can use antibodies against the protein of interest, or even those against the common tags fused to the protein, for western blotting (Ito et al., 2016; Ito and Tomita, 2017). Because phosphorylation of a subset of Rab GTPases by LRRK2 can easily be detected by their co-expression followed by Phos-tag SDS-PAGE, these Rabs were verified to be excellent substrates of LRRK2 in cells. The phospho-specific antibodies selective for LRRK2-mediated phosphorylation are also being established, such as anti-phospho-Thr73 Rab10 (Thirstrup et al., 2017; Fan et al., 2018; Lis et al., 2018) or anti-phospho-Ser106 Rab12 (Thirstrup et al., 2017), and further development of such antibodies is awaited.

A noteworthy finding is that most of the pathogenic LRRK2 mutations commonly and potently enhance its activity to phosphorylate Rab GTPases (Steger et al., 2016; Fujimoto et al., 2018; Liu et al., 2018), leading us to hypothesize that Rab hyperphosphorylation may contribute to the pathogenesis of PD. Recent efforts have thus been focused on the elucidation of the role of phosphorylation of substrate Rabs, especially Rab8 and Rab10, in the physiological and pathological contexts. It has been shown that pathogenic LRRK2 mutations inhibit primary cilia formation that involves Rab8a (Steger et al., 2017) and Rab10 (Dhekne et al., 2018), whereas another group has reported that LRRK2 mutations caused centrosomal defects via phosphorylation of Rab8a (Madero-Perez et al., 2018a) and Rab10 (Ordonez et al., 2019) in dividing cells. Overexpression of both wild-type LRRK2 and Rab29 also caused the same defects (Madero-Perez et al., 2018b). Interestingly, centrosomal cohesion and ciliogenesis were both regulated by their phosphorylation-dependent recruitment to their effector, RILPL1 (Dhekne et al., 2018; Ordonez et al., 2019). Considering that ciliogenesis is controlled by centrosome-mediated regulations, these observations likely converge on a single pathway that could be affected by the hyperphosphorylated Rab8/10.

Regarding the effect of Rab phosphorylation on the endolysosomal system, Rivero-Rios et al. (2019) have reported that G2019S mutant LRRK2 interferes with endolysosomal trafficking of EGFR by impairing Rab8a function. We have reported that LRRK2-mediated phosphorylation of Rab8 and Rab10 functions to maintain lysosomal homeostasis upon overload stresses (Eguchi et al., 2018). That is, when cells are treated with chloroquine, a lysosomotropic agent that induces lysosomal overload by accumulating within its lumen, LRRK2 and Rab8/10 are targeted onto stressed lysosomes, repress lysosomal swellings and facilitates the extracellular secretion of lysosomal contents. These stress responses are positively regulated by LRRK2-mediated phosphorylation of Rab8/10, via recruiting their effectors EHBP1 and EHBP1L1 onto the overloaded lysosomes. We should note that chloroquine treatment induces the extremely diseased conditions in cells that contain swollen lysosomes with undigested materials; although this is different from healthy state, similar cellular pathology can be observed in aged animals (Cuervo and Dice, 2000).

The latter finding is different from the rest of above-mentioned observations in two contexts; first, the lysosomal overload is regulated by endogenous wild-type LRRK2, whereas other defects are induced by the pathogenic LRRK2 mutations or by co-overexpression of wild-type LRRK2 with Rab29. This difference may account for the distinct readouts of LRRK2 kinase activity in physiological and pathological conditions, respectively, although the nature of the deleterious effects of the pathogenic mutant LRRK2 on lysosomal overload has not been fully defined. Second, Rab phosphorylation appears to play an inhibitory role in the regulation of centrosomes or endolysosomal EGFR trafficking, whereas the phosphorylation at the same residue plays a promotive role to mitigate the lysosomal overload. These findings are not mutually contradictory; the differences may be explained by the use of different effector proteins (i.e., RILPL1 vs. EHBP1/EHBP1L1) or different subcellular compartment where each Rab is phosphorylated and accumulated (i.e., centrosomes vs. lysosomes).

**FIGURE 2:**
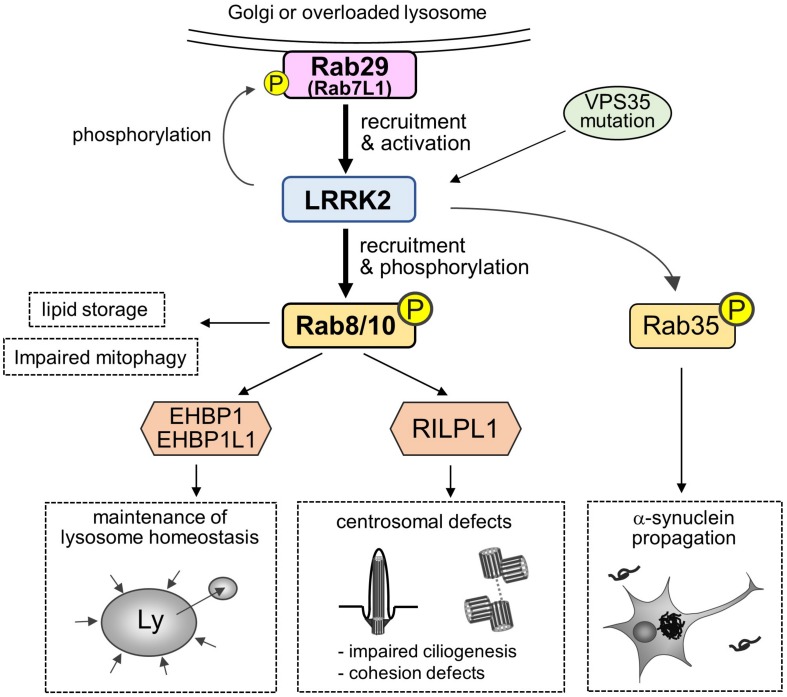
A “Rab29-LRRK2-Rab8/10 cascade” in the pathobiology of LRRK2. Rab29 on Golgi membranes or overloaded lysosomes recruits and activates LRRK2, which in turn causes LRRK2-mediated phosphorylation and recruitment of Rab8/10 at pricentrosomes/centrosomes or lysosomes. This Rab29-LRRK2-Rab8/10 molecular cascade resulted in the modulation of downstream events, such as centrosomal cohesion, ciliogenesis or lysosome maintenance, via recruiting each effector of Rab8/10. LRRK2 can also phosphorylate Rab29 to regulate *trans*-Golgi organization, and VPS35 mutation results in the activation of LRRK2. Other downstream events include Rab8a-mediated promotion of lipid storage, Rab10-mediated mitophagy regulation and Rab35-mediated promotion of α-synuclein propagation.

In addition, there are also intriguing studies reporting the possible readouts of phosphorylation of substrate Rab GTPases by LRRK2, such as the promotion of lipid storage (Yu et al., 2018), *trans*-Golgi organization (Fujimoto et al., 2018), impaired mitophagy (Wauters et al., 2019), and α-synuclein propagation (Bae et al., 2018). Collectively, the roles and impacts of Rab phosphorylation are being uncovered (Figure 2).

## Relationship Between Rab29 and LRRK2

In contrast to Rab8 and Rab10 that act downstream of LRRK2, another LRRK2 substrate, Rab29 (Rab7L1), appears to function upstream of LRRK2. *Rab29* was originally highlighted in PD research as a gene located within PD risk locus *PARK16* (Satake et al., 2009), and the variants at *PARK16* have been suggested to function coordinately with the common variants at *LRRK2* locus to increase PD risk (MacLeod et al., 2013; Pihlstrom et al., 2015). Importantly, *Rab29* KO mice share the key histological phenotypes of *Lrrk2* KO mice, in terms of the accumulation of enlarged secondary lysosomes in the kidney proximal tubules (Kuwahara et al., 2016). This *in vivo* observation can be explained by our cell-based studies in which Rab29 recruits LRRK2 to the overloaded lysosomes to maintain lysosomal homeostasis (Eguchi et al., 2018). This recruitment by Rab29 is observed at an endogenous level, as knockdown of Rab29 prevented lysosomal localization of endogenous LRRK2. The observation that Rab29 acts upstream of LRRK2 was preceded by other studies showing that Rab29 recruits LRRK2 to the *trans*-Golgi network (TGN) or TGN-derived vesicles (MacLeod et al., 2013; Beilina et al., 2014) where Rab29 normally resides (Helip-Wooley and Thoene, 2004), and potently upregulates the LRRK2 kinase activity (Purlyte et al., 2018). Following this study, Madero-Perez et al. (2018b) have also shown that Rab29 recruits LRRK2 to the Golgi complex and causes centrosomal deficits, although the recruitment of LRRK2 to the Golgi by Rab29 was observed solely under overexpressed conditions. As the above-noted studies commonly showed that the recruitment of LRRK2 by Rab29 in turn results in the recruitment of Rab8 and Rab10 that are phosphorylated by LRRK2, this tandem flow of recruitment may work as the central “Rab29-LRRK2-Rab8/10 cascade” in the LRRK2 pathobiology (Figure 2).

It is not yet clear how Rab29 facilitates the recruitment and activation of LRRK2. Rab29 has been shown to directly bind LRRK2 (MacLeod et al., 2013; Beilina et al., 2014), and the binding site on LRRK2 has been mapped to the N terminus of LRRK2, such as the ankyrin repeats (Purlyte et al., 2018), armadillo repeats (Mcgrath et al., 2019) or HEAT domain that spans these repeats (Beilina et al., 2014). However, GTP-binding activity of Rab29 is unlikely to affect its interaction with LRRK2, although it should be noted that Rab29 is not likely a typical small GTPase; actually, a Rab29 mutant predicted to mimic the GTP-bound state (Q67N) unexpectedly showed low ability to retain GTP (Beilina et al., 2014), and that this mutant is diffusely distributed in the cytoplasm upon overexpression (MacLeod et al., 2013; Wang et al., 2014). A more recent study has shown that wild-type Rab29 poorly binds the nucleotide, is inefficiently prenylated, and is not bound to a guanine nucleotide dissociation inhibitor (GDI) in the cytosol (Gomez et al., 2019). Therefore, although LRRK2 functions under the control of Rab29, it functions independently of the classical Rab GTP/GDP switch mechanism and thus behaves differently from typical Rab effectors. Nonetheless, it has also been shown that GTP binding and membrane association of Rab29 are required for its ability to activate LRRK2 as well as the downstream Rab10 recruitment and phosphorylation (Gomez et al., 2019). Taken together, the Rab29-LRRK2-Rab8/10 cascade is even reminiscent of the so-called “Rab cascade” (Pfeffer, 2017), although LRRK2 is not likely a guanine nucleotide exchange factor (GEF) of Rab8/10.

To gain more insights into the mechanisms of LRRK2 activation, we should pay more attention to the potent upregulation of LRRK2 kinase activity by the pathogenic mutation in *VPS35* (Mir et al., 2018), another causative gene for autosomal-dominant late-onset PD (Vilariño-Güell et al., 2011; Zimprich et al., 2011). VPS35 is a major component of the retromer complex that functions at the step of membrane trafficking from early endosomes to *trans*-Golgi, and the dysfunction in this step results in the defective recycling of mannose 6-phosphate receptor (MPR) that delivers lysosomal components into lysosomes. In addition, a prior study has suggested the tripartite functional connection among Rab29, LRRK2 and VPS35 in the intraneuronal membrane trafficking (MacLeod et al., 2013). Thus, it would be interesting to study the detailed relationship between Rab29 and VPS35, both of which regulate LRRK2 kinase activity and lysosomal functions as upstream factors.

It is also unclear how Rab29 phosphorylation by LRRK2 influences the activity of Rab29 to upregulate LRRK2, although it has been reported that a phosphomimetic mutant Rab29, harboring both T71E and S72E, abolished its activity to activate LRRK2 (Purlyte et al., 2018). Two possibilities are considered from this result: first, the T71E/S72E double mutant is not functional; second, Rab29 phosphorylation by LRRK2 acts as a negative feedback to suppress the prolonged activation of LRRK2. Of note, Rab29 phosphorylation at Ser72 may also influence the *trans*-Golgi morphology (Fujimoto et al., 2018), prompting us to speculate that the direct and indirect outcomes of Rab29 phosphorylation might be involved in the possible cellular roles.

## Relevance to the Disease Mechanisms

The impacts of LRRK2 and its substrate Rab GTPases in the endolysosomal system have also been implicated in the pathomechanism of Parkinson’s and related disorders. It has been reported that, in the brains of patients with PD or dementia with Lewy bodies, LRRK2 is abnormally localized to the enlarged granules or vacuoles that correspond to the endolysosomal compartment (Higashi et al., 2009), although the specificity of the antibodies employed in this study has not been fully validated. Biochemical analysis of post-mortem brain tissues demonstrated that the levels of lysosomal proteins LAMP2a and GBA were significantly reduced in patients with LRRK2 mutations (Zhao et al., 2018). In fibroblasts from PD patients harboring the LRRK2 G2019S mutation, late endosomes and lysosomes are morphologically altered or disrupted in a LRRK2 kinase activity-dependent manner (Gomez-Suaga et al., 2014; Hockey et al., 2015). One of these studies showed that the dysregulation of lysosome morphology was dependent on an endolysosomal two-pore channel TPC2, which mediates NAADP-induced Ca^2+^ release from acidic organelles (Hockey et al., 2015). Since other studies have provided evidence of an increased LRRK2 kinase activity in idiopathic PD patients (Fraser et al., 2016; Di Maio et al., 2018), LRRK2 kinase-mediated dysregulation of the endolysosomes may be a common event in the pathophysiology of PD.

However, the involvement of LRRK2-mediated phosphorylation of Rab GTPases, such as Rab8 or Rab10, and lysosomes in relation PD remains largely unclear. Bae et al. (2018) have shown that, in cell culture, nematode and rodent models of PD, LRRK2-mediated phosphorylation of Rab35 regulates the propagation of α-synuclein, although they have not systematically analyzed other Rab GTPases involved in this step. They also provided suggestive evidence that the impaired trafficking of α-synuclein to lysosomes may underlie the observed effects. The pathogenic role of Rab35 was also suggested in the previous report showing that the overexpression of Rab35 phosphomutants (T72A, T72D) induced the neurotoxicity in primary cortical neurons and *in vivo* (Jeong et al., 2018), although we should be cautious about the validity of the use of phosphomutants. Furthermore, another study has reported that the protein level of Rab35 was increased in the substantia nigra of transgenic mice expressing pathogenic LRRK2 (R1441C, G2019S), as well as in the serum samples from PD patients (Chiu et al., 2016). This study additionally demonstrated that Rab35 overexpression increased the aggregation and secretion of α-synuclein in SH-SY5Y cells. Collectively, it would be plausible to nominate Rab35 as a promising candidate Rab GTPase regulating α-synuclein pathology downstream of LRRK2 (Figure 2).

However, the PD-related pathogenic role of other LRRK2 substrates, such as Rab8 and Rab10, has not been fully clarified. As Rab8/10 phosphorylation participates in the regulation of lysosome morphology and release, it would be reasonable to speculate that hyperphosphorylated Rab8/10 modulates the α-synuclein dynamics (clearance, aggregation or propagation) by affecting the maintenance of lysosomes. Indeed, endolysosomal system has been strongly implicated in the α-synuclein (Desplats et al., 2009; Vidyadhara et al., 2019), and it has been shown that endogenous expression of mutant LRRK2 in neurons caused the disruption of lysosomal morphology as well as the increase of α-synuclein insolubility and release via its kinase activity (Schapansky et al., 2018).

## Conclusion

Ever since LRRK2 has been identified as a major PD gene, much effort has been directed toward unraveling the cellular roles of LRRK2. It is now evident that LRRK2 is a multifaceted protein in a variety of tissues and cells, including immune and nervous systems. Particularly, the altered morphology or function of endolysosomes by LRRK2 are frequently described in the studies using immune-related cells, such as macrophages. In other words, LRRK2-mediated endolysosomal regulation may have critical role(s) in the proper execution of immune and phagocytic responses. For example, LRRK2 has been shown to regulate the efficient clearance of certain pathogens, such as *Listeria monocytogenes*, *Salmonella Typhimurium* and *Mycobacterium tuberculosis* (Zhang et al., 2015; Liu et al., 2017; Hartlova et al., 2018; Shutinoski et al., 2019), which may be explained by the altered regulation of phagolysosomes by LRRK2 in the course of innate immune responses. The action of LRRK2 may also cover the adaptive immunity, because antigen presentation by macrophages or dendritic cells is mediated at least in part by the lysosome-related organelle called MHC class II compartment (MIIC), and *LRRK2* has been identified as a risk gene for systemic lupus erythematosus (SLE), a representative autoimmune disorder (Zhang et al., 2017). Further studies will clarify the most important readout of LRRK2 function and dysfunction around endolysosomes, especially *in vivo*.

Compared with the cellular roles of LRRK2, those of substrate Rab GTPases are yet to be characterized. It is easy to speculate that the phosphorylation of substrate Rab mediates the endolysosomal membrane trafficking downstream of LRRK2, and it would be feasible to assess the contribution of each Rab GTPase. The effect of each phosphorylated Rab on PD pathomechanism would be another big issue to be resolved. Given that Rab phosphorylation is enhanced by pathogenic LRRK2 mutations and that Rab is a critical regulator of membrane transport, it is plausible to hypothesize that the perturbation of intracellular trafficking by hyperphosphorylation of Rab GTPases may eventually cause neurodegeneration.

Based on the above views, pharmaceutical companies are now conducting or planning clinical studies of LRRK2 kinase inhibitors for the treatment or prevention of PD^1^. Denali Therapeutics, a leading company developing these inhibitors, has reported that the secretion of a lysosomal lipid bis(monoacylglycerol) phosphate (BMP) into urine and cerebrospinal fluid (CSF) was significantly decreased in humans treated with a LRRK2 inhibitor (source: Denali Therapeutics slide deck^2^). This result is consistent with another line of evidence that LRRK2 KO mice and LRRK2 inhibitor-treated monkeys exhibited decreases in urinary BMP (Fuji et al., 2015), and that urinary BMP was elevated in humans carrying LRRK2 G2019S mutation (Alcalay et al., 2019). These results support the notion that LRRK2 kinase activity contributes to the increased lysosomal secretion, shedding light on the importance of lysosomes in LRRK2 pathobiology. As LRRK2 may be an optimal target for the modification of pathway leading to PD, accelerating the basic research further in various experimental settings and in humans will pave the way toward the establishment of new, cutting-edge strategies to overcome PD.

## Author Contributions

TK conceived and wrote the article. TI provided intellectual input for the contents and edited the manuscript.

## Conflict of Interest

The authors declare that the research was conducted in the absence of any commercial or financial relationships that could be construed as a potential conflict of interest.

## Footnotes

1 https://clinicaltrials.gov/

2 https://denalitherapeutics.com/uploads/documents/events/DNL201_MJFF_PDTx_Presentation.pdf

## References

[B1] Alcalay R. N. Hsieh F. Tengstrand E. Padmanabhan S. Baptista M. Kehoe C. (2019). Higher urine bis(Monoacylglycerol)phosphate levels in LRRK2 G2019S mutation carriers: implications for therapeutic development. *Mov. Disord.* 35 134–141. 10.1002/mds.27818 31505072 PMC6981003

[B2] Alegre-Abarrategui J. Christian H. Lufino M. M. Mutihac R. Venda L. L. Ansorge O. (2009). LRRK2 regulates autophagic activity and localizes to specific membrane microdomains in a novel human genomic reporter cellular model. *Hum. Mol. Genet.* 18 4022–4034. 10.1093/hmg/ddp346 19640926 PMC2758136

[B3] Bae E. J. Kim D. K. Kim C. Mante M. Adame A. Rockenstein E. (2018). LRRK2 kinase regulates alpha-synuclein propagation via RAB35 phosphorylation. *Nat. Commun.* 9:3465. 10.1038/s41467-018-05958-z 30150626 PMC6110743

[B4] Baptista M. A. Dave K. D. Frasier M. A. Sherer T. B. Greeley M. Beck M. J. (2013). Loss of leucine-rich repeat kinase 2 (LRRK2) in rats leads to progressive abnormal phenotypes in peripheral organs. *PLoS One* 8:e80705. 10.1371/journal.pone.0080705 24244710 PMC3828242

[B5] Barrett J. C. Hansoul S. Nicolae D. L. Cho J. H. Duerr R. H. Rioux J. D. (2008). Genome-wide association defines more than 30 distinct susceptibility loci for Crohn’s disease. *Nat. Genet.* 40 955–962. 10.1038/ng.175 18587394 PMC2574810

[B6] Beilina A. Rudenko I. N. Kaganovich A. Civiero L. Chau H. Kalia S. K. (2014). Unbiased screen for interactors of leucine-rich repeat kinase 2 supports a common pathway for sporadic and familial Parkinson disease. *Proc. Natl. Acad. Sci. U.S.A.* 111 2626–2631. 10.1073/pnas.1318306111 24510904 PMC3932908

[B7] Berger Z. Smith K. A. Lavoie M. J. (2010). Membrane localization of LRRK2 is associated with increased formation of the highly active LRRK2 dimer and changes in its phosphorylation. *Biochemistry* 49 5511–5523. 10.1021/bi100157u 20515039 PMC2987719

[B8] Biskup S. Moore D. J. Celsi F. Higashi S. West A. B. Andrabi S. A. (2006). Localization of LRRK2 to membranous and vesicular structures in mammalian brain. *Ann. Neurol.* 60 557–569. 10.1002/ana.21019 17120249

[B9] Biskup S. Moore D. J. Rea A. Lorenz-Deperieux B. Coombes C. E. Dawson V. L. (2007). Dynamic and redundant regulation of LRRK2 and LRRK1 expression. *BMC Neurosci.* 8:102. 10.1186/1471-2202-8-102 18045479 PMC2233633

[B10] Blauwendraat C. Heilbron K. Vallerga C. L. Bandres-Ciga S. Von Coelln R. Pihlstrom L. (2019). Parkinson’s disease age at onset genome-wide association study: defining heritability, genetic loci, and alpha-synuclein mechanisms. *Mov. Disord.* 34 866–875. 10.1002/mds.27659 30957308 PMC6579628

[B11] Boddu R. Hull T. D. Bolisetty S. Hu X. Moehle M. S. Daher J. P. (2015). Leucine-rich repeat kinase 2 deficiency is protective in rhabdomyolysis-induced kidney injury. *Hum. Mol. Genet.* 24 4078–4093. 10.1093/hmg/ddv147 25904107 PMC4476452

[B12] Bosgraaf L. Van Haastert P. J. (2003). Roc, a Ras/GTPase domain in complex proteins. *Biochim. Biophys. Acta* 1643 5–10. 10.1016/j.bbamcr.2003.08.008 14654223

[B13] Chang D. Nalls M. A. Hallgrímsdóttir I. B. Hunkapiller J. Van Der Brug M. Cai F. (2017). A meta-analysis of genome-wide association studies identifies 17 new Parkinson’s disease risk loci. *Nat. Genet.* 49 1511–1516. 10.1038/ng.3955 28892059 PMC5812477

[B14] Chiu C. C. Yeh T. H. Lai S. C. Weng Y. H. Huang Y. C. Cheng Y. C. (2016). Increased Rab35 expression is a potential biomarker and implicated in the pathogenesis of Parkinson’s disease. *Oncotarget* 7 54215–54227. 10.18632/oncotarget.11090 27509057 PMC5342336

[B15] Cuervo A. M. Dice J. F. (2000). When lysosomes get old. *Exp. Gerontol.* 35 119–131. 10.1016/s0531-5565(00)00075-9 10767573

[B16] de Duve C. (2005). The lysosome turns fifty. *Nat. Cell Biol.* 7 847–849. 10.1038/ncb0905-847 16136179

[B17] Dell’Angelica E. C. Mullins C. Caplan S. Bonifacino J. S. (2000). Lysosome-related organelles. *FASEB J.* 14 1265–1278. 10.1096/fj.14.10.1265 10877819

[B18] Desplats P. Lee H. J. Bae E. J. Patrick C. Rockenstein E. Crews L. (2009). Inclusion formation and neuronal cell death through neuron-to-neuron transmission of alpha-synuclein. *Proc. Natl. Acad. Sci. U.S.A.* 106 13010–13015. 10.1073/pnas.0903691106 19651612 PMC2722313

[B19] Dhekne H. S. Yanatori I. Gomez R. C. Tonelli F. Diez F. Schule B. (2018). A pathway for Parkinson’s disease LRRK2 kinase to block primary cilia and Sonic hedgehog signaling in the brain. *Elife* 7:e40202. 10.7554/eLife.40202 30398148 PMC6219843

[B20] Di Maio R. Hoffman E. K. Rocha E. M. Keeney M. T. Sanders L. H. De Miranda B. R. (2018). LRRK2 activation in idiopathic Parkinson’s disease. *Sci. Transl. Med.* 10:eaar5429. 10.1126/scitranslmed.aar5429 30045977 PMC6344941

[B21] Do C. B. Tung J. Y. Dorfman E. Kiefer A. K. Drabant E. M. Francke U. (2011). Web-based genome-wide association study identifies two novel loci and a substantial genetic component for Parkinson’s disease. *PLoS Genet.* 7:e1002141. 10.1371/journal.pgen.1002141 21738487 PMC3121750

[B22] Dodson M. W. Leung L. K. Lone M. Lizzio M. A. Guo M. (2014). Novel ethyl methanesulfonate (EMS)-induced null alleles of the *Drosophila* homolog of LRRK2 reveal a crucial role in endolysosomal functions and autophagy in vivo. *Dis. Model. Mech.* 7 1351–1363. 10.1242/dmm.017020 25288684 PMC4257004

[B23] Dodson M. W. Zhang T. Jiang C. Chen S. Guo M. (2012). Roles of the *Drosophila* LRRK2 homolog in Rab7-dependent lysosomal positioning. *Hum. Mol. Genet.* 21 1350–1363. 10.1093/hmg/ddr573 22171073 PMC3284123

[B24] Eguchi T. Kuwahara T. Sakurai M. Komori T. Fujimoto T. Ito G. (2018). LRRK2 and its substrate Rab GTPases are sequentially targeted onto stressed lysosomes and maintain their homeostasis. *Proc. Natl. Acad. Sci. U.S.A.* 115 E9115–E9124. 10.1073/pnas.1812196115 30209220 PMC6166828

[B25] Fan Y. Howden A. J. M. Sarhan A. R. Lis P. Ito G. Martinez T. N. (2018). Interrogating Parkinson’s disease LRRK2 kinase pathway activity by assessing Rab10 phosphorylation in human neutrophils. *Biochem. J.* 475 23–44. 10.1042/BCJ20170803 29127255 PMC5748842

[B26] Fraser K. B. Rawlins A. B. Clark R. G. Alcalay R. N. Standaert D. G. Liu N. (2016). Ser(P)-1292 LRRK2 in urinary exosomes is elevated in idiopathic Parkinson’s disease. *Mov. Disord.* 31 1543–1550. 10.1002/mds.26686 27297049 PMC5053851

[B27] Fuji R. N. Flagella M. Baca M. Baptista M. A. Brodbeck J. Chan B. K. (2015). Effect of selective LRRK2 kinase inhibition on nonhuman primate lung. *Sci. Transl. Med.* 7:273ra215. 10.1126/scitranslmed.aaa3634 25653221

[B28] Fujimoto T. Kuwahara T. Eguchi T. Sakurai M. Komori T. Iwatsubo T. (2018). Parkinson’s disease-associated mutant LRRK2 phosphorylates Rab7L1 and modifies trans-Golgi morphology. *Biochem. Biophys. Res. Commun.* 495 1708–1715. 10.1016/j.bbrc.2017.12.024 29223392

[B29] Gardet A. Benita Y. Li C. Sands B. E. Ballester I. Stevens C. (2010). LRRK2 is involved in the IFN-gamma response and host response to pathogens. *J. Immunol.* 185 5577–5585. 10.4049/jimmunol.1000548 20921534 PMC3156100

[B30] Gomez R. C. Wawro P. Lis P. Alessi D. R. Pfeffer S. R. (2019). Membrane association but not identity is required for LRRK2 activation and phosphorylation of Rab GTPases. *J. Cell Biol.* 218 4157–4170. 10.1083/jcb.201902184 31624137 PMC6891090

[B31] Gómez-Suaga P. Luzón-Toro B. Churamani D. Zhang L. Bloor-Young D. Patel S. (2012). Leucine-rich repeat kinase 2 regulates autophagy through a calcium-dependent pathway involving NAADP. *Hum. Mol. Genet.* 21 511–525. 10.1093/hmg/ddr481 22012985 PMC3259011

[B32] Gomez-Suaga P. Rivero-Rios P. Fdez E. Blanca Ramirez M. Ferrer I. Aiastui A. (2014). LRRK2 delays degradative receptor trafficking by impeding late endosomal budding through decreasing Rab7 activity. *Hum. Mol. Genet.* 23 6779–6796. 10.1093/hmg/ddu395 25080504

[B33] Hanafusa H. Yagi T. Ikeda H. Hisamoto N. Nishioka T. Kaibuchi K. (2019). LRRK1 phosphorylation of Rab7 at S72 links trafficking of EGFR-containing endosomes to its effector RILP. *J. Cell Sci.* 132:jcs228809. 10.1242/jcs.22880 31085713

[B34] Hartlova A. Herbst S. Peltier J. Rodgers A. Bilkei-Gorzo O. Fearns A. (2018). LRRK2 is a negative regulator of *Mycobacterium tuberculosis* phagosome maturation in macrophages. *EMBO J.* 37:e98694. 10.15252/embj.201798694 29789389 PMC6003659

[B35] Hatano T. Kubo S. Imai S. Maeda M. Ishikawa K. Mizuno Y. (2007). Leucine-rich repeat kinase 2 associates with lipid rafts. *Hum. Mol. Genet.* 16 678–690. 10.1093/hmg/ddm013 17341485

[B36] Helip-Wooley A. Thoene J. G. (2004). Sucrose-induced vacuolation results in increased expression of cholesterol biosynthesis and lysosomal genes. *Exp. Cell Res.* 292 89–100. 10.1016/j.yexcr.2003.09.003 14720509

[B37] Henry A. G. Aghamohammadzadeh S. Samaroo H. Chen Y. Mou K. Needle E. (2015). Pathogenic LRRK2 mutations, through increased kinase activity, produce enlarged lysosomes with reduced degradative capacity and increase ATP13A2 expression. *Hum. Mol. Genet.* 24 6013–6028. 10.1093/hmg/ddv314 26251043

[B38] Herzig M. C. Kolly C. Persohn E. Theil D. Schweizer T. Hafner T. (2011). LRRK2 protein levels are determined by kinase function and are crucial for kidney and lung homeostasis in mice. *Hum. Mol. Genet.* 20 4209–4223. 10.1093/hmg/ddr348 21828077 PMC3188995

[B39] Higashi S. Moore D. J. Yamamoto R. Minegishi M. Sato K. Togo T. (2009). Abnormal localization of leucine-rich repeat kinase 2 to the endosomal-lysosomal compartment in lewy body disease. *J. Neuropathol. Exp. Neurol.* 68 994–1005. 10.1097/NEN.0b013e3181b44ed8 19680143 PMC2768772

[B40] Hinkle K. M. Yue M. Behrouz B. Dachsel J. C. Lincoln S. J. Bowles E. E. (2012). LRRK2 knockout mice have an intact dopaminergic system but display alterations in exploratory and motor co-ordination behaviors. *Mol. Neurodegener.* 7:25. 10.1186/1750-1326-7-25 22647713 PMC3441373

[B41] Hockey L. N. Kilpatrick B. S. Eden E. R. Lin-Moshier Y. Brailoiu G. C. Brailoiu E. (2015). Dysregulation of lysosomal morphology by pathogenic LRRK2 is corrected by TPC2 inhibition. *J. Cell Sci.* 128 232–238. 10.1242/jcs.164152 25416817 PMC4294771

[B42] Hopfner F. Schulte E. C. Mollenhauer B. Bereznai B. Knauf F. Lichtner P. (2013). The role of SCARB2 as susceptibility factor in Parkinson’s disease. *Mov. Disord.* 28 538–540. 10.1002/mds.25349 23408458

[B43] Hui K. Y. Fernandez-Hernandez H. Hu J. Schaffner A. Pankratz N. Hsu N. Y. (2018). Functional variants in the LRRK2 gene confer shared effects on risk for Crohn’s disease and Parkinson’s disease. *Sci. Transl. Med.* 10:eaai7795. 10.1126/scitranslmed.aai7795 29321258 PMC6028002

[B44] Ito G. Katsemonova K. Tonelli F. Lis P. Baptista M. A. Shpiro N. (2016). Phos-tag analysis of Rab10 phosphorylation by LRRK2: a powerful assay for assessing kinase function and inhibitors. *Biochem. J.* 473 2671–2685. 10.1042/BCJ20160557 27474410 PMC5003698

[B45] Ito G. Tomita T. (2017). Rab10 phosphorylation detection by LRRK2 activity using SDS-PAGE with a phosphate-binding Tag. *J. Vis. Exp.* 130: e56688. 10.3791/56688 29286425 PMC5755567

[B46] Iwaki H. Blauwendraat C. Leonard H. L. Liu G. Maple-Grodem J. Corvol J. C. (2019). Genetic risk of Parkinson disease and progression: an analysis of 13 longitudinal cohorts. *Neurol. Genet.* 5:e348. 10.1212/NXG.0000000000000348 31404238 PMC6659137

[B47] Jeong G. R. Jang E. H. Bae J. R. Jun S. Kang H. C. Park C. H. (2018). Dysregulated phosphorylation of Rab GTPases by LRRK2 induces neurodegeneration. *Mol. Neurodegener.* 13:8. 10.1186/s13024-018-0240-1 29439717 PMC5811984

[B48] Kalia L. V. Lang A. E. Hazrati L.-N. N. Fujioka S. Wszolek Z. K. Dickson D. W. (2015). Clinical correlations with Lewy body pathology in LRRK2-related Parkinson disease. *JAMA Neurol.* 72 100–105. 10.1001/jamaneurol.2014.2704 25401511 PMC4399368

[B49] Khan N. L. Jain S. Lynch J. M. Pavese N. Abou-Sleiman P. Holton J. L. (2005). Mutations in the gene LRRK2 encoding dardarin (PARK8) cause familial Parkinson’s disease: clinical, pathological, olfactory and functional imaging and genetic data. *Brain* 128(Pt 12) 2786–2796. 10.1093/brain/awh667 16272164

[B50] Kim K. S. Marcogliese P. C. Yang J. Callaghan S. M. Resende V. Abdel-Messih E. (2018). Regulation of myeloid cell phagocytosis by LRRK2 via WAVE2 complex stabilization is altered in Parkinson’s disease. *Proc. Natl. Acad. Sci. U.S.A.* 115 E5164–E5173. 10.1073/pnas.1718946115 29760073 PMC5984500

[B51] Kinoshita E. Kinoshita-Kikuta E. Takiyama K. Koike T. (2006). Phosphate-binding tag, a new tool to visualize phosphorylated proteins. *Mol. Cell. Proteomics* 5 749–757. 10.1074/mcp.t500024-mcp200 16340016

[B52] Kuwahara T. Inoue K. D’agati V. D. Fujimoto T. Eguchi T. Saha S. (2016). LRRK2 and RAB7L1 coordinately regulate axonal morphology and lysosome integrity in diverse cellular contexts. *Sci. Rep.* 6:29945. 10.1038/srep29945 27424887 PMC4947924

[B53] Lawrence R. E. Zoncu R. (2019). The lysosome as a cellular centre for signalling, metabolism and quality control. *Nat. Cell Biol.* 21 133–142. 10.1038/s41556-018-0244-7 30602725

[B54] Li X. Tan Y. C. Poulose S. Olanow C. W. Huang X. Y. Yue Z. (2007). Leucine-rich repeat kinase 2 (LRRK2)/PARK8 possesses GTPase activity that is altered in familial Parkinson’s disease R1441C/G mutants. *J. Neurochem.* 103 238–247. 17623048 10.1111/j.1471-4159.2007.04743.x PMC2827244

[B55] Lill C. M. Roehr J. T. Mcqueen M. B. Kavvoura F. K. Bagade S. Schjeide B. M. (2012). Comprehensive research synopsis and systematic meta-analyses in Parkinson’s disease genetics: the PDGene database. *PLoS Genet.* 8:e1002548. 10.1371/journal.pgen.1002548 22438815 PMC3305333

[B56] Lis P. Burel S. Steger M. Mann M. Brown F. Diez F. (2018). Development of phospho-specific Rab protein antibodies to monitor in vivo activity of the LRRK2 Parkinson’s disease kinase. *Biochem. J.* 475 1–22. 10.1042/BCJ20170802 29127256 PMC5748839

[B57] Liu W. Liu X. Li Y. Zhao J. Liu Z. Hu Z. (2017). LRRK2 promotes the activation of NLRC4 inflammasome during *Salmonella* Typhimurium infection. *J. Exp. Med.* 214 3051–3066. 10.1084/jem.20170014 28821568 PMC5626397

[B58] Liu Z. Bryant N. Kumaran R. Beilina A. Abeliovich A. Cookson M. R. (2018). LRRK2 phosphorylates membrane-bound Rabs and is activated by GTP-bound Rab7L1 to promote recruitment to the trans-Golgi network. *Hum. Mol. Genet.* 27 385–395. 10.1093/hmg/ddx410 29177506 PMC5886198

[B59] MacLeod D. Dowman J. Hammond R. Leete T. Inoue K. Abeliovich A. (2006). The familial Parkinsonism gene LRRK2 regulates neurite process morphology. *Neuron* 52 587–593. 10.1016/j.neuron.2006.10.008 17114044

[B60] MacLeod D. A. Rhinn H. Kuwahara T. Zolin A. Di Paolo G. Mccabe B. D. (2013). RAB7L1 interacts with LRRK2 to modify intraneuronal protein sorting and Parkinson’s disease risk. *Neuron* 77 425–439. 10.1016/j.neuron.2012.11.033 23395371 PMC3646583

[B61] Madero-Perez J. Fdez E. Fernandez B. Lara Ordonez A. J. Blanca Ramirez M. Gomez-Suaga P. (2018a). Parkinson disease-associated mutations in LRRK2 cause centrosomal defects via Rab8a phosphorylation. *Mol. Neurodegener.* 13:3. 10.1186/s13024-018-0235-y 29357897 PMC5778812

[B62] Madero-Perez J. Fernandez B. Lara Ordonez A. J. Fdez E. Lobbestael E. Baekelandt V. (2018b). RAB7L1-mediated relocalization of LRRK2 to the Golgi complex causes centrosomal deficits via RAB8A. *Front. Mol. Neurosci.* 11:417. 10.3389/fnmol.2018.00417 30483055 PMC6243087

[B63] Maekawa T. Kubo M. Yokoyama I. Ohta E. Obata F. (2010). Age-dependent and cell-population-restricted LRRK2 expression in normal mouse spleen. *Biochem. Biophys. Res. Commun.* 392 431–435. 10.1016/j.bbrc.2010.01.041 20079710

[B64] Manzoni C. Mamais A. Dihanich S. Abeti R. Soutar M. P. M. Plun-Favreau H. (2013). Inhibition of LRRK2 kinase activity stimulates macroautophagy. *Biochim. Biophys. Acta* 1833 2900–2910. 10.1016/j.bbamcr.2013.07.020 23916833 PMC3898616

[B65] Martin I. Kim J. Lee B. Kang H. Xu J.-C. Jia H. (2014). Ribosomal protein s15 phosphorylation mediates LRRK2 Neurodegeneration in Parkinson’s disease. *Cell* 157 472–485. 10.1016/j.cell.2014.01.064 24725412 PMC4040530

[B66] Matta S. Van Kolen K. Da Cunha R. Van Den Bogaart G. Mandemakers W. Miskiewicz K. (2012). Lrrk2 controls an EndoA phosphorylation cycle in synaptic endocytosis. *Neuron* 75 1008–1021. 10.1016/j.neuron.2012.08.022 22998870

[B67] Mcgrath E. Waschbusch D. Baker B. M. Khan A. R. (2019). LRRK2 binds to the Rab32 subfamily in a GTP-dependent manner via its armadillo domain. *Small GTPases* 1–14. 10.1080/21541248.2019.1666623 31552791 PMC7849779

[B68] Michelakakis H. Xiromerisiou G. Dardiotis E. Bozi M. Vassilatis D. Kountra P. M. (2012). Evidence of an association between the scavenger receptor class B member 2 gene and Parkinson’s disease. *Mov. Disord.* 27 400–405. 10.1002/mds.24886 22223122

[B69] Mir R. Tonelli F. Lis P. Macartney T. Polinski N. K. Martinez T. N. (2018). The Parkinson’s disease VPS35[D620N] mutation enhances LRRK2-mediated Rab protein phosphorylation in mouse and human. *Biochem. J.* 475 1861–1883. 10.1042/BCJ20180248 29743203 PMC5989534

[B70] Nalls M. A. Pankratz N. Lill C. M. Do C. B. Hernandez D. G. Saad M. (2014). Large-scale meta-analysis of genome-wide association data identifies six new risk loci for Parkinson’s disease. *Nat. Genet.* 46 989–993. 10.1038/ng.3043 25064009 PMC4146673

[B71] Ness D. Ren Z. Gardai S. Sharpnack D. Johnson V. J. Brennan R. J. (2013). Leucine-rich repeat kinase 2 (LRRK2)-deficient rats exhibit renal tubule injury and perturbations in metabolic and immunological homeostasis. *PLoS One* 8:e66164. 10.1371/journal.pone.0066164 23799078 PMC3682960

[B72] Ordonez A. J. L. Fernandez B. Fdez E. Romo-Lozano M. Madero-Perez J. Lobbestael E. (2019). RAB8, RAB10 and RILPL1 contribute to both LRRK2 kinase-mediated centrosomal cohesion and ciliogenesis deficits. *Hum. Mol. Genet.* 28 3552–3568. 10.1093/hmg/ddz201 31428781 PMC6927464

[B73] Orenstein S. J. Kuo S. H. Tasset I. Arias E. Koga H. Fernandez-Carasa I. (2013). Interplay of LRRK2 with chaperone-mediated autophagy. *Nat. Neurosci.* 16 394–406. 10.1038/nn.3350 23455607 PMC3609872

[B74] Paisan-Ruiz C. Jain S. Evans E. W. Gilks W. P. Simon J. Van Der Brug M. (2004). Cloning of the gene containing mutations that cause PARK8-linked Parkinson’s disease. *Neuron* 44 595–600. 10.1016/j.neuron.2004.10.023 15541308

[B75] Pfeffer S. R. (2017). Rab GTPases: master regulators that establish the secretory and endocytic pathways. *Mol. Biol. Cell* 28 712–715. 10.1091/mbc.E16-10-0737 28292916 PMC5349778

[B76] Pihlstrom L. Rengmark A. Bjornara K. A. Dizdar N. Fardell C. Forsgren L. (2015). Fine mapping and resequencing of the PARK16 locus in Parkinson’s disease. *J. Hum. Genet.* 60 357–362. 10.1038/jhg.2015.34 25855069

[B77] Plowey E. D. Cherra S. J. Liu Y. J. Chu C. T. (2008). Role of autophagy in G2019S−LRRK2−associated neurite shortening in differentiated SH−SY5Y cells. *J. Neurochem.* 105 1048–1056. 10.1111/j.1471-4159.2008.05217.x 18182054 PMC2361385

[B78] Pu J. Guardia C. M. Keren-Kaplan T. Bonifacino J. S. (2016). Mechanisms and functions of lysosome positioning. *J. Cell Sci.* 129 4329–4339. 10.1242/jcs.196287 27799357 PMC5201012

[B79] Purlyte E. Dhekne H. S. Sarhan A. R. Gomez R. Lis P. Wightman M. (2018). Rab29 activation of the Parkinson’s disease-associated LRRK2 kinase. *EMBO J.* 37 1–18. 10.15252/embj.201798099 29212815 PMC5753036

[B80] Ramirez A. Heimbach A. Gründemann J. Stiller B. Hampshire D. Cid P. L. (2006). Hereditary parkinsonism with dementia is caused by mutations in ATP13A2, encoding a lysosomal type 5 P-type ATPase. *Nat. Genet.* 38 1184–1191. 10.1038/ng1884 16964263

[B81] Reczek D. Schwake M. Schroder J. Hughes H. Blanz J. Jin X. (2007). LIMP-2 is a receptor for lysosomal mannose-6-phosphate-independent targeting of beta-glucocerebrosidase. *Cell* 131 770–783. 10.1016/j.cell.2007.10.018 18022370

[B82] Rivero-Rios P. Romo-Lozano M. Madero-Perez J. Thomas A. P. Biosa A. Greggio E. (2019). The G2019S variant of leucine-rich repeat kinase 2 (LRRK2) alters endolysosomal trafficking by impairing the function of the GTPase RAB8A. *J. Biol. Chem.* 294 4738–4758. 10.1074/jbc.RA118.005008 30709905 PMC6442034

[B83] Rothaug M. Zunke F. Mazzulli J. R. Schweizer M. Altmeppen H. Lullmann-Rauch R. (2014). LIMP-2 expression is critical for beta-glucocerebrosidase activity and alpha-synuclein clearance. *Proc. Natl. Acad. Sci. U.S.A.* 111 15573–15578. 10.1073/pnas.1405700111 25316793 PMC4217458

[B84] Saftig P. Klumperman J. (2009). Lysosome biogenesis and lysosomal membrane proteins: trafficking meets function. *Nat. Rev. Mol. Cell Biol.* 10 623–635. 10.1038/nrm2745 19672277

[B85] Satake W. Nakabayashi Y. Mizuta I. Hirota Y. Ito C. Kubo M. (2009). Genome-wide association study identifies common variants at four loci as genetic risk factors for Parkinson’s disease. *Nat. Genet.* 41 1303–1307. 10.1038/ng.485 19915576

[B86] Schapansky J. Khasnavis S. Deandrade M. P. Nardozzi J. D. Falkson S. R. Boyd J. D. (2018). Familial knockin mutation of LRRK2 causes lysosomal dysfunction and accumulation of endogenous insoluble alpha-synuclein in neurons. *Neurobiol. Dis.* 111 26–35. 10.1016/j.nbd.2017.12.005 29246723 PMC5803451

[B87] Schapansky J. Nardozzi J. D. Felizia F. Lavoie M. J. (2014). Membrane recruitment of endogenous LRRK2 precedes its potent regulation of autophagy. *Hum. Mol. Genet.* 23 4201–4214. 10.1093/hmg/ddu138 24682598 PMC4103671

[B88] Shutinoski B. Hakimi M. Harmsen I. E. Lunn M. Rocha J. Lengacher N. (2019). Lrrk2 alleles modulate inflammation during microbial infection of mice in a sex-dependent manner. *Sci. Transl. Med.* 11:eaas9292. 10.1126/scitranslmed.aas9292 31554740

[B89] Simón-Sánchez J. Schulte C. Bras J. M. Sharma M. Gibbs R. J. Berg D. (2009). Genome-wide association study reveals genetic risk underlying Parkinson’s disease. *Nat. Genet.* 41 1308–1312. 10.1038/ng.487 19915575 PMC2787725

[B90] Steger M. Diez F. Dhekne H. S. Lis P. Nirujogi R. S. Karayel O. (2017). Systematic proteomic analysis of LRRK2-mediated Rab GTPase phosphorylation establishes a connection to ciliogenesis. *Elife* 6:e31012. 10.7554/eLife.31012 29125462 PMC5695910

[B91] Steger M. Tonelli F. Ito G. Davies P. Trost M. Vetter M. (2016). Phosphoproteomics reveals that Parkinson’s disease kinase LRRK2 regulates a subset of Rab GTPases. *Elife* 5:e12813. 10.7554/eLife.12813 26824392 PMC4769169

[B92] Thirstrup K. Dachsel J. C. Oppermann F. S. Williamson D. S. Smith G. P. Fog K. (2017). Selective LRRK2 kinase inhibition reduces phosphorylation of endogenous Rab10 and Rab12 in human peripheral mononuclear blood cells. *Sci. Rep.* 7:10300. 10.1038/s41598-017-10501-z 28860483 PMC5578959

[B93] Tong Y. Giaime E. Yamaguchi H. Ichimura T. Liu Y. Si H. (2012). Loss of leucine-rich repeat kinase 2 causes age-dependent bi-phasic alterations of the autophagy pathway. *Mol. Neurodegener.* 7:2. 10.1186/1750-1326-7-2 22230652 PMC3296570

[B94] Tong Y. Yamaguchi H. Giaime E. Boyle S. Kopan R. Kelleher R. J. (2010). Loss of leucine-rich repeat kinase 2 causes impairment of protein degradation pathways, accumulation of alpha-synuclein, and apoptotic cell death in aged mice. *Proc. Natl. Acad. Sci. U.S.A.* 107 9879–9884. 10.1073/pnas.1004676107 20457918 PMC2906862

[B95] Vidyadhara D. J. Lee J. E. Chandra S. S. (2019). Role of the endolysosomal system in Parkinson’s disease. *J. Neurochem.* 150 487–506. 10.1111/jnc.14820 31287913 PMC6707858

[B96] Vilariño-Güell C. Wider C. Ross O. A. Dachsel J. C. Kachergus J. M. Lincoln S. J. (2011). VPS35 Mutations in Parkinson disease. *Am. J. Hum. Genet.* 89 162–167. 21763482 10.1016/j.ajhg.2011.06.001 PMC3135796

[B97] Vitte J. Traver S. Maués De Paula A. Lesage S. Rovelli G. Corti O. (2010). Leucine-rich repeat kinase 2 is associated with the endoplasmic reticulum in dopaminergic neurons and accumulates in the core of Lewy bodies in Parkinson disease. *J. Neuropathol. Exp. Neurol.* 69 959–972. 10.1097/NEN.0b013e3181efc01c 20720502

[B98] Wang S. Ma Z. Xu X. Wang Z. Sun L. Zhou Y. (2014). A role of Rab29 in the integrity of the trans-Golgi network and retrograde trafficking of mannose-6-phosphate receptor. *PLoS One* 9:e96242. 10.1371/journal.pone.0096242 24788816 PMC4008501

[B99] Wauters F. Cornelissen T. Imberechts D. Martin S. Koentjoro B. Sue C. (2019). LRRK2 mutations impair depolarization-induced mitophagy through inhibition of mitochondrial accumulation of RAB10. *Autophagy* 16 203–222. 10.1080/15548627.2019.1603548 30945962 PMC6984591

[B100] West A. B. Moore D. J. Biskup S. Bugayenko A. Smith W. W. Ross C. A. (2005). Parkinson’s disease-associated mutations in leucine-rich repeat kinase 2 augment kinase activity. *Proc. Natl. Acad. Sci. U.S.A.* 102 16842–16847. 10.1073/pnas.0507360102 16269541 PMC1283829

[B101] Yu M. Arshad M. Wang W. Zhao D. Xu L. Zhou L. (2018). LRRK2 mediated Rab8a phosphorylation promotes lipid storage. *Lipids Health Dis.* 17:34. 10.1186/s12944-018-0684-x 29482628 PMC5828482

[B102] Zhang F. R. Huang W. Chen S. M. Sun L. D. Liu H. Li Y. (2009). Genomewide association study of leprosy. *N. Engl. J. Med.* 361 2609–2618. 20018961 10.1056/NEJMoa0903753

[B103] Zhang Q. Pan Y. Yan R. Zeng B. Wang H. Zhang X. (2015). Commensal bacteria direct selective cargo sorting to promote symbiosis. *Nat. Immunol.* 16 918–926. 10.1038/ni.3233 26237551

[B104] Zhang Y. M. Zhou X. J. Cheng F. J. Qi Y. Y. Hou P. Zhao M. H. (2017). Autophagy-related gene LRRK2 is likely a susceptibility gene for systemic lupus erythematosus in northern Han Chinese. *Oncotarget* 8 13754–13761. 10.18632/oncotarget.14631 28099919 PMC5355135

[B105] Zhao Y. Perera G. Takahashi-Fujigasaki J. Mash D. C. Vonsattel J. P. G. Uchino A. (2018). Reduced LRRK2 in association with retromer dysfunction in post-mortem brain tissue from LRRK2 mutation carriers. *Brain* 141 486–495. 10.1093/brain/awx344 29253086 PMC5837795

[B106] Zimprich A. Benet-Pagès A. Struhal W. Graf E. Eck S. H. Offman M. N. (2011). A mutation in VPS35, encoding a subunit of the retromer complex, causes late-onset parkinson disease. *Am. J. Hum. Genet.* 89 168–175. 10.1016/j.ajhg.2011.06.008 21763483 PMC3135812

[B107] Zimprich A. Biskup S. Leitner P. Lichtner P. Farrer M. Lincoln S. (2004). Mutations in LRRK2 cause autosomal-dominant parkinsonism with pleomorphic pathology. *Neuron* 44 601–607. 10.1016/j.neuron.2004.11.005 15541309

